# Supplementary material for: Impact of Socio-Economic Status on Accessibility of Dog Training Classes
Source: Animals (Basel). 2019 Oct 22;9(10):849. doi: 10.3390/ani9100849 (PMC6826670; doi:10.3390/ani9100849)
Supplement: Supplementary file 1 [file animals-09-00849-s001.pdf]

Supplementary material- Course Content

Introduction to Dog School– 3 week Course Content

Introduction to Dog School Learning Objectives

1. Give owners key information about their dog's needs and behaviour to prevent the development of undesired behaviours.

2. Help owners to identify contexts in which their dog shows signs of anxiety, fear or frustration, and those in which they are able to relax or enjoy themselves.

3. Demonstrate reward based methods for training and provide a safe space for owners to practice – particularly training behaviours that are important for basic control in family life: loose lead walking and recall, and developing these to be maintained in different environments and with distractions.

4. Encourage dogs to start learning to decide for themselves to show the appropriate behaviour in different contexts (e.g. settle when owner isn't giving the dog attention or is busy).

5. Pick up any individual concerns or early onset problems that owners have and provide specific advice to address these.

Key messages for dog owners (12 things your dog wants you to know)

1. Observe and understand your dog's body language, especially to recognise signs of anxiety, fear and frustration.

2. Identify situations where your dog is worried or over stimulated so that you can control their exposure to those environments, and allow them to learn a new response.

3. Understand that dogs are often worried about being alone. Recognise signs that indicate they might be distressed, and help them relax when separated.

4. Understand how dogs are always learning about their environment and that their current behaviour is influenced by their previous experiences.

5. Enable dogs to learn for themselves which behaviours works to achieve the good things in life (during training but also in everyday activities and interactions).

6. Be positive and consistent so it is easy for your dog to learn about you.

7. Ensure that your dog learns to settle down and expect quiet times when they are with people.

8. Understand what your dog values so you can reward them for behaviours you want.

9. Prevent your dog from becoming worried about people approaching the things they value, such as their food and toys.

10. Understand dog social behaviour, so that you can prevent problems developing.

11. Provide your dog with opportunities to show normal dog behaviours.

12. Understand how dogs develop strong expectations and can become frustrated when these are not met. Know how to respond appropriately to prevent frustration related problems developing.

Class set-up for weeks two and three

- Set-up to provide each owner and dog with a safe place/area without direct contact with other dogs. The areas are separated with retractable barriers. Owners need to be introduced to their individual areas, and the dogs start to learn to settle in those locations. A maximum of 6 dogs per class.

Week One

Introductory session

Owners are invited to attend a presentation as a group without their dogs for the first session. The session starts with a welcome and introduction to Dog School, an explanation of what to expect from the two subsequent classes and how they can prepare for them.

Owners to be given a copy of the appropriate booklet for their dog's age – either puppy, adolescent or adult/rescue.

The session then introduces some of the key messages of the classes, including the concepts of:

- Developing a positive relationship with their dog.
- Importance of social behaviour in dogs, and recognising signs of anxiety, fear, frustration as well as overexcitement.
- Introduction to how dogs learn:
  - Importance of rewards in training and learning new behaviours.
  - Setting up the environment so that dogs can learn for themselves which behaviours work to achieve the good things in life.
  - Understanding how dogs can learn behaviours we don't like, as well as those we do.
- Helping dogs learn to cope with being alone.
- People being consistent in interactions with their dogs so it is easy for dogs to learn.
- The importance of providing dogs with the opportunity to show normal dog behaviours.

Week Two

1. Come in and settle. Introductions and welcomes.
2. **KEY MESSAGE:** Brief discussion about how we will be using food in classes (to reward good behaviours). Check for dietary intolerances. Discuss why some dogs may be worried about being approached whilst eating – and the common signs to look for (standing over their food, freezing, moving away, growling). Check for any of these issues before providing Kongs™ where suitable. Discuss how to swap the Kong for treats instead of taking it.

3. **ACTIVITY 1: Settle.** What, where, when, how. Give 5 minutes to practice quietly. Keep practising as you talk over the next key message.
4. **KEY MESSAGE:** Basic information on training – associations between events that occur close together in time, importance of timing reinforcements, concept of a marker to identify the correct behaviour.
5. **ACTIVITY 2: Use marker to teach** sit (or a stand for those dogs that find it uncomfortable or painful to sit). Start to move on to down for those that sit quickly. Start with the basics of learning the behaviour– lure the dog into position, mark the correct behaviour, fade the lure. Discuss the use– many like to use sit (or down/stand) as a 'polite' behaviour. Discuss introducing sit for common situations where the dog may otherwise show excitable behaviours (i.e. saying hello to people, going through doorways, at pavements, getting dinner, lead on/off). First the dog is prompted; if you are consistent the dog will then start to offer the behaviour without being asked. The reward can be environmental (crossing road, getting through door, going off lead).
6. **Settle.** Go back to teaching the settle from scratch now that dogs and owners have had some time to adjust since arrival. Start to phase out Kongs™/settle aids where appropriate.
7. **KEY MESSAGE: Attention as a resource**. Importance of being consistent and understanding how differences in owner response impacts on dogs. Discuss in relation to excitement, fear, frustration and emotional conflict. Provide examples of where things can go wrong, and right in light of attention seeking behaviours. For example jumping up and stealing.
8. **ACTIVITY 3: Four paws on the floor**. Discuss why dogs jump up, and how to teach an alternate behaviour – owners start with their own dog reinforcing relaxed behaviour on approach. See 4 paws on the floor exercise for instructions. Discuss other examples of where consistency is important whilst practising. Discuss that dogs may become frustrated when they find that their normal attention seeking behaviour stops working and try harder – stress the importance of not responding to the dog whilst they are showing inappropriate behaviour to avoid reinforcing it, but to reward the right behaviours as much as possible to help them learn quickly.
9. **Settle.**
10. **KEY MESSAGE: Introduce loose lead walking**. Discuss different training equipment – what is recommended, needs for different dogs. Explain problems associated with negative methods. Discuss why dogs pull on the lead and how to teach them to walk with a loose lead using reward based methods. Discuss how loose lead walking is a really great way of teaching dogs that in order to get where they want to go, they need to walk calmly and pay attention to their owner, rather than rushing forwards.
11. **ACTIVITY 4: Loose lead walking** – demonstrate and practice.
12. **Settle.** Try without Kongs™.
13. **KEY MESSAGE: Recall.** Discuss the importance of teaching a reliable recall, and that this is a behaviour that needs reinforcing throughout the dog's life. Discuss why it is not uncommon for owners to notice that their adolescent dogs start to struggle with recall, and start venturing further afield. Discuss the common mistakes– for example only calling the dog back before putting them on the lead, at the end of the walk or when they have been 'naughty'. Encourage owners to become the most exciting thing on their dogs walk, and use only reward based training methods to ensure that their dogs are excited to come back to them. Discuss the use of different rewards, and finding out what their dog likes best. Talk about

predictability – importance of varying the walk so that their dog has to keep an eye on them (for example by walking in new places, changing their route or simply changing direction regularly). Set dogs up for success by calling when the owner knows they are likely to succeed, and using a long line attached to a fixed, flat harness during training, to keep the dog safe and ensure training is a success.

14. **ACTIVTY 5: Recall exercise.** May need to introduce appropriate harnesses for any dogs that have not got their own – in which case this first session may be a 'new equipment' introduction for some individuals. Can introduce concept of distraction for dogs who are ready.
15. **Settle** – check for any problems / questions. Hand out homework for this week – settle and recall.
16. Ask people to leave one by one to avoid uncontrolled contact.

<u>Week Three</u>

1. Come in and settle in own areas. Provide Kongs™ only for dogs that need them. Review previous week; identify any difficulties and help problem solve.
2. **KEY MESSAGE: Explain why dogs may be worried by being left alone** and how to identify and manage this. Link in with the uses of settle training. Advise referral for anyone with moderate-severe separation issue.
3. **ACTIVITY 1: Settle.** Try without Kongs™.
4. **KEY MESSAGE: Dealing with chewing and mouthing** – introduce why these behaviours occur and how they may be reinforced / get worse over time. Ask if anyone has problems with these behaviours. Discuss how to deal with these behaviours – making discussion relevant to specific dogs in the class if appropriate.
5. **KEY MESSAGE: Play**. Discuss the importance of providing suitable opportunities for play. Discuss that most young dogs love to play, and providing a suitable play outlet with people can help channel their energy into an appropriate direction, provide a powerful reward for training and help the dog-owner relationship. Discuss rules of the games (i.e. no teeth on skin, no snatching the toy). Include mention of feeding enrichment as well as exercise opportunities and mind games.
6. **ACTIVITY 2: Appropriate play**. Demonstrate how to use toys to engage dogs in play (i.e. tug) – engage with dog and teach them to swap toy for treats. If teeth go onto human, play stops. Control arousal of dog by stopping game before it becomes too intense, and restarting. Discuss the benefits (teaching dogs they can become excited, but can also calm down quickly). If coach prefers, or for dogs who are reluctant to tug play games with treats (scent games, food enrichment, hide and seek etc.).
7. **Settle**
8. **KEY MESSAGE**: Identifying signs of fear and anxiety – why it is important, and what signs to look for. Link in to explaining how handle your dog appropriately, introduce handling exercise.
9. **ACTIVITY 3: Habituation to handling exercise**. Gradual increase in handling, pick out any signs of anxiety or frustration and highlight.
10. **Settle**
11. **KEY MESSAGE: Recap of why some dogs guard** – value of resource and perceived threat of losing it, and why it is important to reduce the value of resources and make sure dogs are

not anxious about people approaching. Explain how to make sure that the dog is not worried about people approaching valued resources. Explain how the Kong™ swapping exercise is a starting point for this. Check again for guarding issues in the class before introducing next exercise.

12. **ACTIVITY 4: Teaching the 'leave'**. Reduce frustration as much as possible during exercise and build up slowly. Stress that the 'leave' is a positive cue. Dogs are rewarded for waiting patiently and taking their attention away from their goal!
13. **KEY MESSAGE: Discuss frustration**. This is the emotion triggered when expectations have not been met, and is more likely to occur when certain needs (i.e. physical/mental stimulation) are not being met. Dogs need to learn how to cope when things don't go the way they expect. It is normal for dogs to respond actively to frustration – by trying harder – or perhaps trying other behaviours such as mouthing or barking in order to reach their goal. It's important that we support dogs by pre-empting situation that may cause frustration and rewarding our dogs for good behaviours instead – such as waiting calmly while we prepare their meals. Don't get angry if your dog reacts in an unwanted manner to feeling frustrated; simply help them learn a better alternative. Ensuring they are kept active in body and mind can help reduce frustration related problems.
14. **ACTIVITY 5: Walking through a doorway** (if environment/weather suitable this could be going out through the door to leave one at a time – waiting for the door to be opened and walking calmly through).
15. **Settle** – check for any problems / questions. Provide homework (leave) and info on how to book courses if owners wish to in future.
16. Ask people to leave one by one to avoid uncontrolled contact.
